# Identification of Multiple Stress Responsive Genes by Sequencing a Normalized cDNA Library from Sea-Land Cotton (*Gossypium barbadense* L.)

**DOI:** 10.1371/journal.pone.0152927

**Published:** 2016-03-31

**Authors:** Bin Zhou, Lin Zhang, Abid Ullah, Xin Jin, Xiyan Yang, Xianlong Zhang

**Affiliations:** National Key Laboratory of Crop Genetic Improvement, Huazhong Agricultural University, Wuhan, Hubei 430070, P. R. China; USDA-ARS-SRRC, UNITED STATES

## Abstract

**Background:**

Plants often face multiple stresses including drought, extreme temperature, salinity, nutrition deficiency and biotic stresses during growth and development. All the stresses result in a series of physiological and metabolic reactions and then generate reversible inhibition of metabolism and growth and can cause seriously irreversible damage, even death. At each stage of cotton growth, environmental stress conditions pose devastating threats to plant growth and development, especially yield and quality. Due to the complex stress conditions and unclear molecular mechanisms of stress response, there is an urgent need to explore the mechanisms of cotton response against abiotic stresses.

**Methodology and Principal Findings:**

A normalized cDNA library was constructed using *Gossypium barbadense* Hai-7124 treated with different stress conditions (heat, cold, salt, drought, potassium and phosphorus deficit and *Verticillium dahliae* infection). Random sequencing of this library generated 6,047 high-quality expressed sequence tags (ESTs). The ESTs were clustered and assembled into 3,135 uniESTs, composed of 2,497 contigs and 638 singletons. The blastx results demonstrated 2,746 unigenes showing significant similarity to known genes, 74 uniESTs displaying significant similarity to genes of predicted proteins, and 315 uniESTs remain uncharacterized. Functional classification unveiled the abundance of uniESTs in binding, catalytic activity, and structural molecule activity. Annotations of the uniESTs by the plant transcription factor database (PlantTFDB) and Plant Stress Protein Database (PSPDB) disclosed that transcription factors and stress-related genes were enriched in the current library. The expression of some transcription factors and specific stress-related genes were verified by RT-PCR under various stress conditions.

**Conclusions/Significance:**

Annotation results showed that a huge number of genes respond to stress in our study, such as *MYB-related*, *C2H2*, *FAR1*, *bHLH*, *bZIP*, *MADS*, and *mTERF*. These results will improve our knowledge of stress tolerance in cotton. In addition, they are also helpful in discovering candidate genes related to stress tolerance. The publicly available ESTs from *G*. *barbadense* are a valuable genomic resource that will facilitate further molecular study and breeding of stress-tolerant cotton.

## Introduction

In recent years, because of the dramatic changes in climate and environment, natural disasters occur frequently, posing serious threats to the growth and yield of crops worldwide [1]. It is estimated that a third of the cultivated area of the world suffers from chronically inadequate supplies of water. It is also reported that arid and subarid regions occupy half of the total land area in China [2]. Additionally, there are more than 950 million hectares of saline and alkaline land throughout the world [3]. The plantation of early spring plants and harvest of late autumn plants are also restricted by cold damage [4]. Thus, understanding the molecular mechanisms governing plant stress response and tolerance in plants is vital for increasing the stress response and tolerance of crops.

The tolerance of plants to stress is a complex trait controlled by multiple factors. There is a common genetic mechanism in stress tolerance induced by different factors for example drought, salinity, high and low temperature, featuring membrane permeability, active oxygen balance, osmotic regulation, and hormone signal transduction [5–9]. In last few years, a batch of genes concern to stress response has been identified through mutant screening, homologous comparison, and differential screening of transcriptome and cDNA libraries, combined with biological information and genetic transformation of cotton [10–13]; some of these techniques have been proved impressive in developing tolerance to various abiotic stresses in cotton [14–17]. Stress-related genes products can be classified into two classes. The first class includes regulatory molecules, such as proteins concerned with the regulation of signal transduction and downstream stress-responsive genes. These contain numerous transcription factors, protein phosphatase, protein kinases, and other signaling molecules such as calcium receptors. The second class includes downstream protein molecules for instance water channel proteins, sugar and proline transporters, chaperones, osmotin, late embryogenesis abundant (LEA) proteins, antifreeze proteins, and detoxification enzymes, as well as various proteases [18,19].

Cotton is native to tropical and subtropical zones and is relatively resistant to stress. However, its long growth period makes it suffer from a variety of abiotic stresses that reduce production or quality. For example, cotton is sensitive to high temperature during the blooming period; extreme temperature not only inhibits the yield and quality but also seriously affects the normal seedling growth, which causes serious anther abortion and boll shedding [20]. During the reproductive growth stage, yield reduction and fiber quality compromises are inescapable when drought stress conditions override the plant’s protective mechanisms [21]. Cotton is also susceptible to salt and cold stresses during seed germination [22]. During the seeding and seed germination stages, cotton plants often encounter cold waves [23]. Therefore, improving the comprehensive tolerance of cotton to numerous stresses for instance drought, salt, and extreme temperature is very fruitful and vital for cotton breeding.

With an increasing number of studies on molecular mechanisms of stress gene expression regulation and signal transduction in plants and the progress of cotton genomic sequencing, large-scale sequencing projects involving expressed sequence tags (ESTs) for different cotton species have been undertaken by several laboratories [24–28]. Up to September 10, 2015, there were 508,731 ESTs deposited in the dbESTs of NCBI GenBank (http://www.ncbi.nlm.nih.gov/dbEST/dbEST_summary.html) from the *Gossypium* species, covering 337,811 ESTs from *G*. *hirsutum*, 64,798 ESTs from *G*. *arboreum*, 63,577 ESTs from *G*. *raimondii*, and 39,115 ESTs from *G*. *barbadense*. In spite of the importance of the gene resources of *G*. *barbadense*, few EST resources were deposited for this species compared with those of *G*. *hirsutum*, *G*. *arboreum*, and *G*. *raimondii*.

In this study, genomics approaches were applied to explore the transcriptional regulation of *G*. *barbadense* in response to various stress conditions (heat, cold, salt, drought, potassium and phosphorus deficit and *Verticillium dahliae* infection). This paper presents the experiments conducted for sequencing a normalized library of *G*. *barbadense* Hai-7124 (treated by various stresses). Random sequencing generated 6,047 high-quality ESTs, which were then assembled into 3,135 uniESTs, made up of 638 contigs and 2,497 singletons. Based on sequence similarity and Gene Ontology (GO) annotations, the unique sequences were determined as putative functions with small molecules and oxidoreductase enriched. Moreover, putative genes concerned with stress tolerance and transcription factors were also identified in response to stress of *G*. *barbadense*. Contributing to the study of stress resistance mechanisms of *G*. *barbadense*, the dadasets are supposed to prop up valuable resources for comparative genomic studies of *Gossypium* species.

## Results

### Construction of individual and normalized cDNA libraries

Six individual libraries of *G*. *barbadense* cv. Hai-7124 (tolerance to stress and superior properties of fiber quality) treated by different stress factors (heat, cold, salt, drought, potassium and phosphorus deficit and *Verticillium dahliae* infection) were constructed (S1 Table). Randomly selected clones were amplified with M13 forward and reverse to identify the length of inserts. The plasmids from the six individual libraries were mixed and hybridized with genomic DNA of Hai-7124 to generate the normalized cDNA library. Double digestion enzymes were used to release the inserts of the normalized library of randomly selected clones with *EcoR*I and *BamH*I; the length of the inserts ranged largely from 0.8 to 1.5 kb and reached 1.15kb in average (S2 Table).

### Generation and Assembly of ESTs

A total of 7,125 randomly picked EST clones were sequenced from the 5’-end, producing a total of 6,047 high-quality ESTs (84.9%) with quality control for a high confidence base call (Q20) after elimination of no inserts or short insert clones (100 bp cut-off) (S1 Fig). The average length of remaining ESTs was 654 bp, ranged from 100 to 1,260 bp. All of these sequences were submitted to GenBank with accession number of 7124_EST000001- 7124_EST006533. The ESTs were assembled into 3,135 uniESTs, composed of 638 (20.4%) contigs with an average length of 856 bp and 2,497 (79.6%) singletons with an average length of 669 bp. The largest proportion of uniESTs was 700–799 bp in length (788, 25.1%). The detailed information of these ESTs was shown in Table 1.

**Table 1:** EST sequence and assembly statistics.

Parameter	Value
Total number of assembled sequences	6,047
Number of contigs	638
Average number of ESTs in contigs	5.6
Number of singletons	2,497
Number of uniESTs	3,135
Average length of uniESTs (bp)	707
Average length of contigs (bp)	856
Average length of singletons (bp)	669
Longest length of unique sequences (bp)	2,106

**Fig 1:**
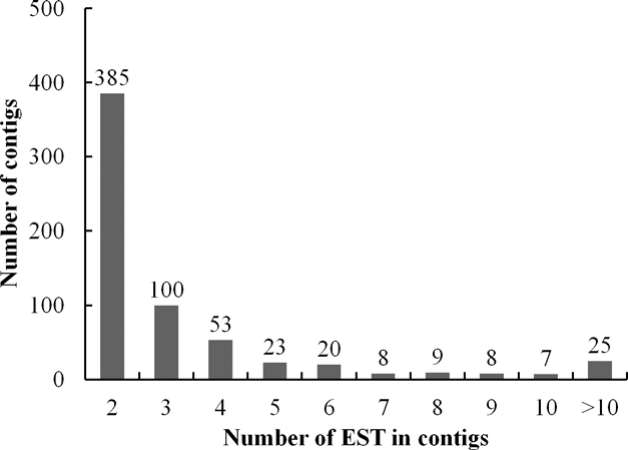
Distribution of 638 contigs based on the number of clustered ESTs.

ESTs per contig averaged at 5.6 in number, while the highest number reached 874. Between 2 and 10 ESTs were assembled into each contig: 60.3% were generated with 2 ESTs, 15.7% with 3 ESTs, and the remaining 24% with 4 or more transcripts (Fig 1), recommending that the cDNA library was normalized well for generating uniESTs. S3 Table (≥ 10 EST copies contained each of these clusters and represented 30.0% of the total number of ESTs obtained) shows the most abundantly expressed genes, encoding key stress proteins, for examples, MLP-like proteins (CO000105, CO000083, and CO000013, including 63 ESTs), peroxidase (CO000085 and CO000042, including 32 ESTs), alcohol dehydrogenase-like 1-like (CO000348, including 19 ESTs), glutathione S transferase (CO000109, including 15 ESTs), important enzymes for photosynthesis, such as chlorophyll A/B binding protein 1 (CO000002 and CO000334, including 49 ESTs), photosystem II subunit (CO000025, CO000006, and CO000113, including 45 ESTs), and ribulose bisphosphate carboxylase (CO000085 and CO000026, including 38 ESTs), disease resistance-responsive family protein (CO000269, including 12 ESTs), and some important transporters (CO000018, CO000084, and CO000159, including 899 ESTs). There were also some novel sequences without function annotations, such as CO000012 and CO000028.

### UniEST Annotations

**Fig 2:**
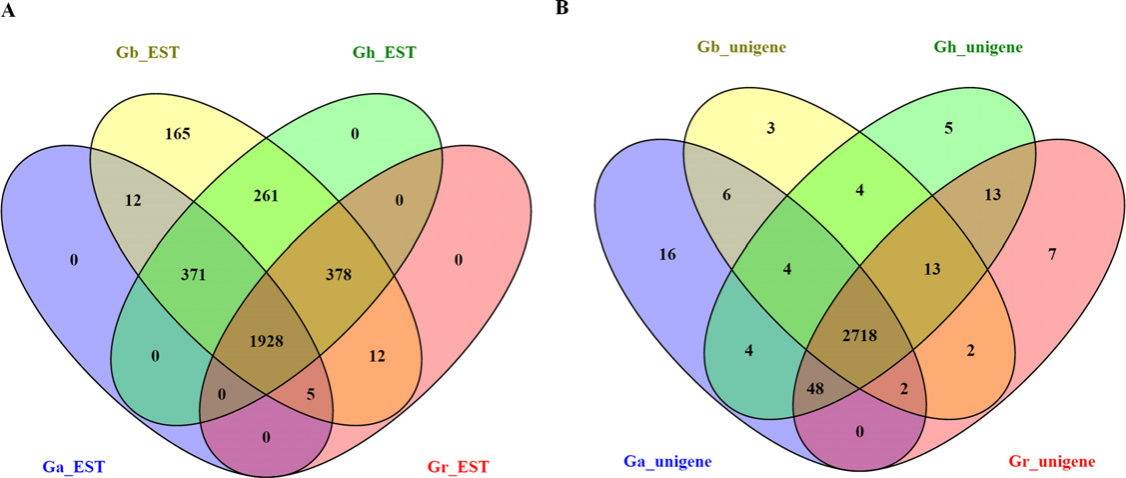
The distribution of uniESTs in four reference cotton species obtained using four different cotton species EST databases and unigenes from different cotton subspecies genome databases. Venn diagrams showing the uniESTs in each of the four different cotton species EST databases (A) or unigenes from different cotton subspecies genome databases (B), with the overlapping regions corresponding to the number of uniESTs present in more than one species. The central region corresponds to the uniESTs present in all four species.

The 3,135 uniESTs were mapped to several reference sequences (ESTs of different cotton species downloaded from GenBank: *G*. *hirsutum*, 337,811; *G*. *arboreum*, 64,798; *G*. *raimondii*, 63,577; and *G*. *barbadense*, 39,115; (http://www.ncbi.nlm.nih.gov/dbEST/dbEST_summary.html)). The distribution of the uniEST best blastx hits was 3,132 (99.9%) in *G*. *barbadense*, 2,938 (93.7%) in *G*. *hirsutum*, 2,323 (74.1%) in *G*. *raimondii* and 2,316 (73.9%) in *G*. *arboreum*, (E-value ≤ 10^−5^) (Fig 2A and S4 Table). A total of 3,132 (99.9%) uniESTs had similarity with one or more species, and 1,928 (61.5%) uniESTs were shared by four cotton species. On the other hand, none of the uniESTs was shared by only one species among *G*. *raimondii* (D-genome), *G*. *arboreum* (A-genome) and *G*. *hirsutum* (AD-genome) (Fig 2A). Of the unidentified uniESTs, there were 3 (0. 1%), 197 (6.3%), 812 (25.9%), and 819 (26.1%), in *G*. *barbadense*, *G*. *hirsutum*, *G*. *raimondii*, *and G*. *arboreum*, respectively, which might be regarded as novel or specific genes that respond to different stress treatments.

The 3,135 uniESTs were also blasted with several reference sequences (unigenes from different cotton species: *G*. *arboreum*, 75,594; *G*. *raimondii*, 77, 267; *G*. *hirsutum*, 288,299; and *G*. *barbadense*, 80,876). The distribution of the unigene best blastx hits was 2,809 (89.6%) in *G*. *hirsutum*, 2,803 (89.4%) in *G*. *raimondii*, 2,798 (89.3%) in *G*. *arboreum* and 2,752 (87.8%) in *G*. *barbadense* (E-value ≤ 10^−5^) (S5 Table). A total of 2,845 (90.7%) unigenes showed similarity with one or more species, and 2,718 (86.7%) unigenes were shared by four cotton species. Nevertheless, of the unigenes shared with only one species, 7, 16 and 5 unigenes were shared with *G*. *raimondii* (D-genome), *G*. *arboreum* (A-genome) and *G*. *hirsutum* (AD-genome), respectively (Fig 2B). However, of the unidentified unigenes, 383 (12.2%), 326 (10.4%), 332 (10.6%), and 337 (10.7%) were considered as novel or specific genes in *G*. *barbadense*, *G*. *hirsutum*, *G*. *raimondii*, and *G*. *arboreum*, respectively, against different stress conditions.

The uniESTs were further searched against the Genbank non-redundant protein database by means of blastx, and overall 2,820 (90.0%) uniESTs presented significant hits (E-value ≤ 10^−5^), of which there were only 2341 uniESTs (74.7% of the total uniESTs) with similarity to known genes and 479 uniESTs (15.3%) with similarity to genes of unknown function at the amino acid level. The remaining 315 uniESTs (10.0%) did not exhibit resemblance to protein sequences deposited in the Nr database. Among the uniESTs, 22 (0.7%) and 162 (5.2%) encoded photosystem-related proteins and ribosomal proteins, respectively, confirming the successfully normalized coverage of the cDNA library (S6 Table).

**Fig 3:**
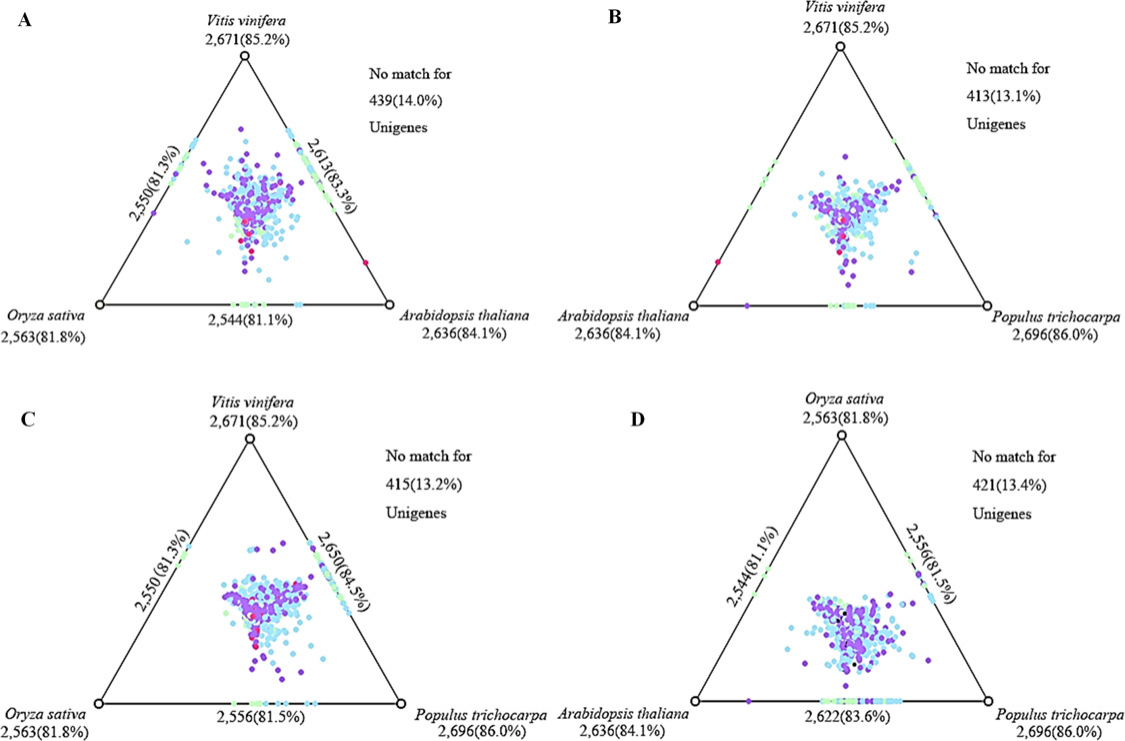
SimiTri profile of the unigenes. The 3,135 uniESTs were blastx searched against the protein sequences of four different species (A, B, C, D) (E-value ≤ 10^−5^). The color was coded based on the highest BLAST score as follows: red > 750; pink > 450; purple > 250; blue > 100, and green <100.

Furthermore, the uniESTs were blast against *Arabidopsis thaliana*, *Oryza sativa*, *Populus trichocarpa*, and *Vitis vinifera* protein sequences using blastx (E-value ≤ 10^−5^). The results showed the following matches: 2,696 (86.0%) in *Populus trichocarpa*, 2,671 (85.2%) in *Vitis vinifera*, 2,636 (84.1%) in *Arabidopsis thaliana* and 2,563 (81.8%) in *Oryza sativa* (S7 Table). The two-dimensional illustration of the relative similarity relationships between the four species is shown using the program SimiTri [29] (Fig 3A–3D).

### Gene ontology (GO) annotation

Functional groups were allocated to the entire uniESTs in terms of GO annotation through BLAST2GO, which was based on comprehensive information with sequence similarity against the Genbank non-redundant protein database [30]. A total of 2,678 (85.4%) uniESTs were divided into one or more ontologies, of which 1,810 (57.7%), 1,885 (60.1%), and 1,722 (54.9%) uniESTs were assigned the GO categories molecular function (MF), biological process (BP), and cellular component (CC), respectively. A total of 1,286 (41.0%) uniESTs were classified into three ontologies (S8 Table).

**Fig 4:**
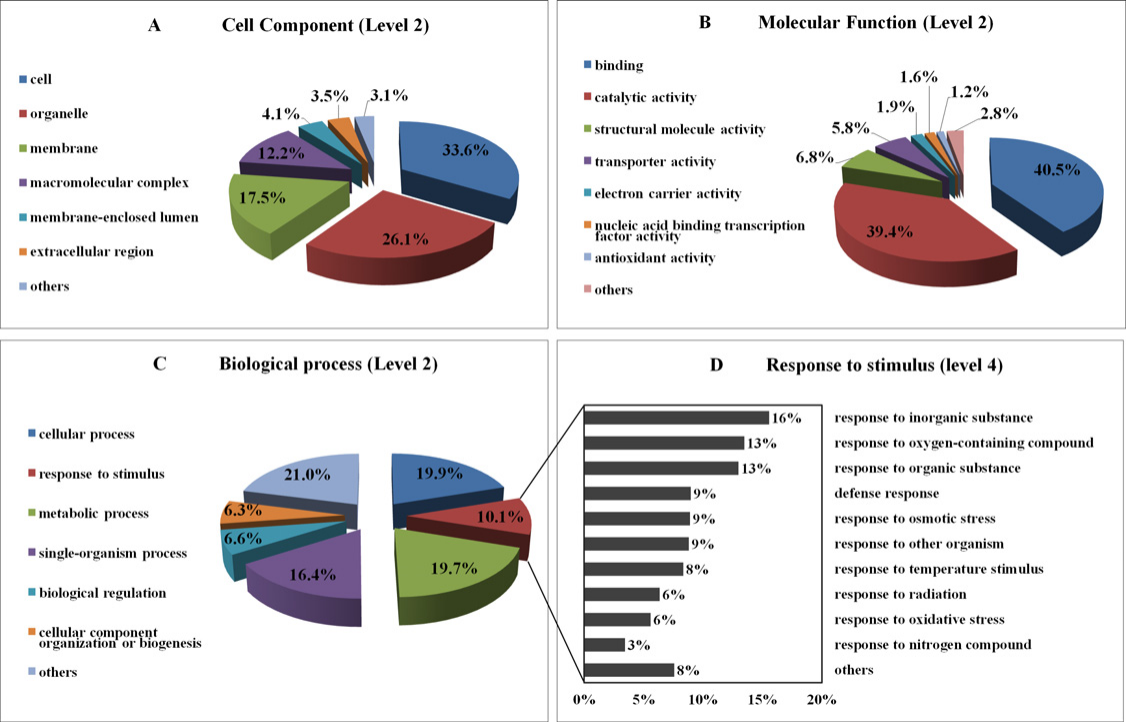
Functional classifications of 2,678 uniESTs that were assigned GO terms. Tortadiagrams represented the second-level GO terms for three categories: cellular component (A), molecular function (B) and biological process (C). The histogram represents the GO term ‘Response to stimulus’ at level 4 (D).

The GO categories MF, BP, and CC fell predominantly into two to four subcategories at the second level (Fig 4A–4D). Among the top 3 GO terms enriched in the CC class (second-level GO terms), terms of cell, organelle, and membrane were accumulated more compared to the other terms (Fig 4A); more category classifications (third-level GO terms) are shown in S2A Fig. In the MF class (second-level GO terms, as shown in the Fig 4B), mostly the GO terms were classified into two groups, i.e. binding (GO:0005488, 40.5%) and catalytic activity (GO:0003824, 39.4%). In the binding category, organic cyclic compound binding (GO:0097159, 14.3%), heterocyclic compound binding (GO: 1901363, 14.3%), ion binding (GO:0043167, 13.7%), and small molecule binding (GO:0036094, 8.8%) ranked the most enriched terms at third level. The highly enriched GO terms in catalytic activity covered transferase activity (GO:0016740, 6.8%), hydrolase activity (GO:0016787, 6.6%) and oxidoreductase activity (GO:0016491, 6.2%) (S2B Fig). For the biological process class, four categories with the GO terms cellular process (GO:0009987, 19.9%), metabolic process (GO:0008152, 19.7%), single-organism process (GO:0044699, 16.4%) and response to stimulus (GO:0050896, 10.1%) were dominantly enriched, which accounted for 66.1% of the assigned uniESTs (Fig 4C); more category classifications at the third level for the category biological process are shown in S2C Fig. The level 4 response to stimulus category mainly consists of ‘response to inorganic substance’, ‘response to oxygen-containing compound’, ‘defense response’, ‘response to osmotic stress’, ‘response to other organism’, ‘response to temperature stimulus’ and others (Fig 4D), which included most stress responses from among our previous treatments.

### KEGG pathway assignment

In addition, KEGG pathway assignments were made to the uniESTs using KOBAS2.0 (KEGG Orthology Based Annotation System) based on comprehensive information with sequence similarity [31,32]. The results showed annotations for 1,903 uniESTs (60.7%) and an enrichment for the category ‘Biosynthesis of amino acids’ (63, 3.3%), followed by the categories ‘Carbon metabolism’ (60, 3.2%), ‘Protein processing in endoplasmic reticulum’ (59, 3.1%), ‘Plant hormone signal transduction’ (43, 2.3%), ‘RNA transport’ (41, 2.2%), ‘Huntington’s disease’ (39, 2.0%), ‘Oxidative phosphorylation’ (39, 2.0%), ‘Epstein-Barr virus infection’ (37, 1.9%) and ‘Photosynthesis’ (36, 1.9%) (Table 2).

**Table 2:** The major distribution of the KEGG pathway.

Categories	Input number	Background number	P-Value
Biosynthesis of amino acids	63	99	0.313896346
Carbon metabolism	60	86	0.157454254
Protein processing in endoplasmic reticulum	59	77	0.065163346
Plant hormone signal transduction	43	43	0.007989226
RNA transport	41	93	0.945554498
Huntington's disease	39	72	0.675475423
Oxidative phosphorylation	39	92	0.963353005
Epstein-Barr virus infection	37	63	0.520001548
Photosynthesis	36	55	0.326094054
Plant-pathogen interaction	31	37	0.084042593
Cysteine and methionine metabolism	30	32	0.0398779
Spliceosome	30	101	0.999795385

### Identification of putative transcription factors and stress-related genes

**Fig 5:**
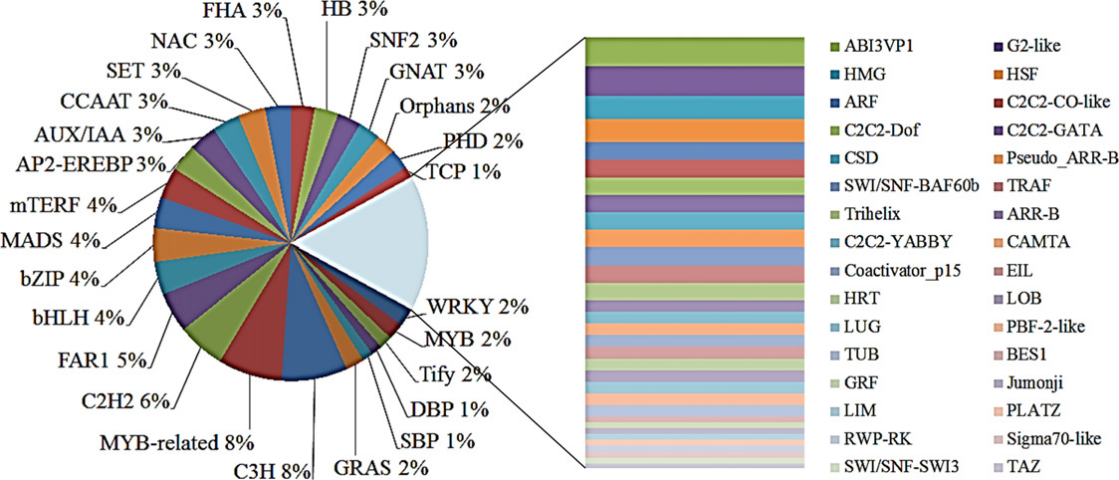
Enrichment of transcription factor genes and classification of TF families. TF genes were classified into TF families using blast against the publicly available Plant Transcription Factor Database (PlnTFDB); the 464 TF genes were classified into 58 TF families. Numbers represent the percentage of TF families out of the 464 TF genes. The classification and annotation of all TF genes with respect to functional categories and transcription factor families are presented in S9 Table.

We used blastx searches against PlantTFDB 3.0, which currently contains 28,193 protein models and 26,184 unique protein sequences, organized in 84 gene families, to identify putative transcription factors [33]. The results revealed 464 (14.8% of uniESTs) matches to PlantTFDB (E-value ≤ 10^−5^) (S9-1 Table). The 464 transcription factors belonged to 58 families. The most abundant transcription factor family was C3H (36, 7.8%) followed by MYB (v-myb myeloblastosis viral oncogene homolog) -related (35, 7.5%), C2H2 (Cys2/His2) (26, 5.6%), FAR1 (FAR-RED IMPAIRED RESPONSE 1) (22, 4.7%), bHLH (basic Helix-Loop-Helix) (18, 3.9%), bZIP (basic region/leucine zipper motif) (18, 3.9%), MADS (MCM1, AGAMOUS, DEFICINES, and SRF) (17, 3.7%), and mTERF (mitochondrial transcription termination factor) (17, 3.7%) (Fig 5 and S9-2 Table). In distribution, the best uniEST blastx hits accounted 21.8% for *O*. *sativa*, 20.7% for *V*. *vinifera*, 17.0% for *P*. *trichocarpa*, 12.1% for *C*. *papaya*, and 9.1% for *Arabidopsis*. The distribution of transcription factor families for the five concerned species is enlisted in S9-3 Table. In comparison to other model species, among the high-frequency transcription factor families, the MYB-related (35, 7.5%), C2H2 (26, 5.6%), FAR1 (22, 4.7%), bHLH (18, 3.9%) and bZIP (18, 3.9%) families showed comparatively higher frequencies in *G*. *barbadense*, whereas the BES1 (BRI1-EMS-SUPPRESSOR 1) (1,0.2%), GRF (GENERAL REGULATORY FACTOR) (1, 0.2%), LIM (LIN-11, Isl-1, and Mec-3) (1, 0.2%), PLATZ (plant AT-rich sequence and zinc-binding protein 1) (1, 0.2%) and TAZ (transcriptional co-activator with PDZ-binding motif) (1, 0.2%) families presented lower frequencies. However, the BSD, LFY, SAP, and E2F-DP families were not detected in our datasets. The bZIP and bHLH families are especially related to intense stress responses.

**Fig 6:**
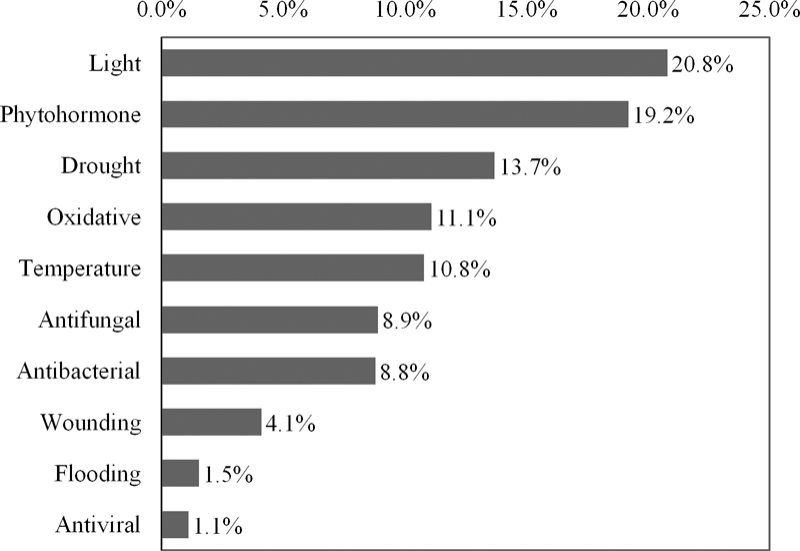
Distribution of 919 stress-related genes out of 3,135 uniESTs.

For annotation of the stress-related genes, the 3,135 uniESTs were blasted to the Plant Stress Protein Database (PSPDB), a web-accessible resource that covers 2,064 manually curated plant stress proteins from a wide array of 134 plant species with 30 different types of biotic and abiotic stresses. A total of 919 uniESTs (29.3%) presented matches with PSPDB (E-value ≤ 10^−5^) (S10 Table) and were annotated as stress-related genes responsive to light (20.8%), phytohormone (19.2%), drought (13.7%), oxidation (11.1%), temperature (10.8%), antifungal (8.9%), antibacterial processes (8.8%), wounding (4.1%), antiviral processes (1.1%), and flooding (1.5%) (Fig 6).

Protein kinases play conserved central roles in metabolic signaling and stress response [34]. The current identified protein kinase family members in the library mainly composed of thirty-seven receptor-like protein kinase (RLK) family members, nine brassinosteroid-signaling kinase family members, eight mitogen-activated protein kinase (MAPK) family members, seven calcineurin B-like calcium sensor interacting protein kinase (CIPK) family members, seven SNF1 (sucrose-non-fermenting 1) kinase family members, six AMP-activated protein kinase (AMPK) family members, fourcalcium-dependent protein kinase (CDPK) family members, and three WNK family members (S11 Table).

Plant hormones such as auxin, cytokinins, gibberellin (GA), abscisic acid (ABA), jasmonic acid (JA), salicylic acid (SA), ethylene (ET), and brassinosteroids (BRs) are small molecules that play significant roles throughout the life span of plants, especially when they encounter adversity [35]. In our research, we found twenty-nine genes responsive to auxin, such as *ARF2 (auxin response factor 2)*, *ARF9 (auxin response factor 9-like)*, *ARF10 (auxin response factor 10)*, *ARF7 (auxin response factor 7)*, *AUX1 (auxin influx transporter 1)*, *ABP1 (auxin-binding protein 1)*, *SAUR-like (auxin-responsive family protein)* and *IAA11 (IAA-responsive protein 11)*; twenty-three genes responsive to ethylene, including *BBM (ap2-like ethylene-responsive transcription factor bbm-like)*, *ERF1 (ethylene-responsive transcription factor 1)*, *ERF2 (ethylene-responsive transcription factor 2)*, *ERF4 (ethylene-responsive transcription factor 4)*, *EIN3 (ethylene-insensitive3)* and *RAP2-4 (ethylene-responsive transcription factor rap2-4-like)*; eleven genes responsive to gibberellin, including *GA4 (gibberellin requiring 4)*, *G2OX8 (gibberellin 2-oxidase 8)*, *G3OX3 (gibberellin 3-oxidase 1)* and *G3OX4 (gibberellin 3-oxidase 4)*; six genes responsive to abscisic acid, including *AREB2 (abscisic acid responsive element-binding factor 2)*, *ABF1 (abscisic acid responsive element-binding factor 1)*, *ABAH1 (abscisic acid 8 -hydroxylase 1-like)* and *PYL9 (pyrabactin resistance 1-like 9)*; six genes responsive to jasmonic acid, namely *JAZ5 (jasmonate-zim-domain protein 5)* and *JAZ12 (jasmonate-zim-domain protein 12)*; four genes responsive to cytokinin, including *LOG3 (lonely guy 3)*, *LOG5 (lonely guy 5)* and *LOG8 (lonely guy 8)*; and two genes responsive to brassinosteroids and salicylic acid, namely *BIN1 (brassinosteroid insensitive 1)*, *BIN2 (brassinosteroid insensitive 2)*, *SAMT (salicylate o-methyltransferase)* and *BSMT1 (both salicylic acid and benzoic acid methyltransferase 1)* (S12 Table). Additionally, fourteen genes were identified as responsive to stresses, such as heat, dehydration and phosphate stress. Examples of these genes are *LEA2 (late embryogenesis-abundant protein 2)*, *LEA5 (late embryogenesis-abundant protein 5)* and *dehydrin*.

Reactive oxygen species (ROS) are oxygen-containing substances of metabolites and their derivatives generated through oxidation in plants directly or indirectly [36,37]. Many ROS could be induced during many environmental stresses, such as hydrogen peroxide, superoxide and hydroxyl radicals [5]. ROS play a vital role in stress signal transduction, but excessive active oxygen oxidation harms the plants. Therefore, plants also produce a series of active oxygen scavenging enzymes, for example, peroxidase (POD), catalase (CAT), superoxide dismutase (SOD), and glutathione S transferase (GST), among others. Moreover, there are other non-enzyme components such as ascorbic acid, glutathione, carotenoids, and flavonoids, among others. [38]. In our study, forty-six redoxin family proteins, twenty-three peroxidases, eleven glutathione S transferases, four superoxide dismutases and one catalase were found (S13 Table).

### RT-PCR analysis

**Fig 7:**
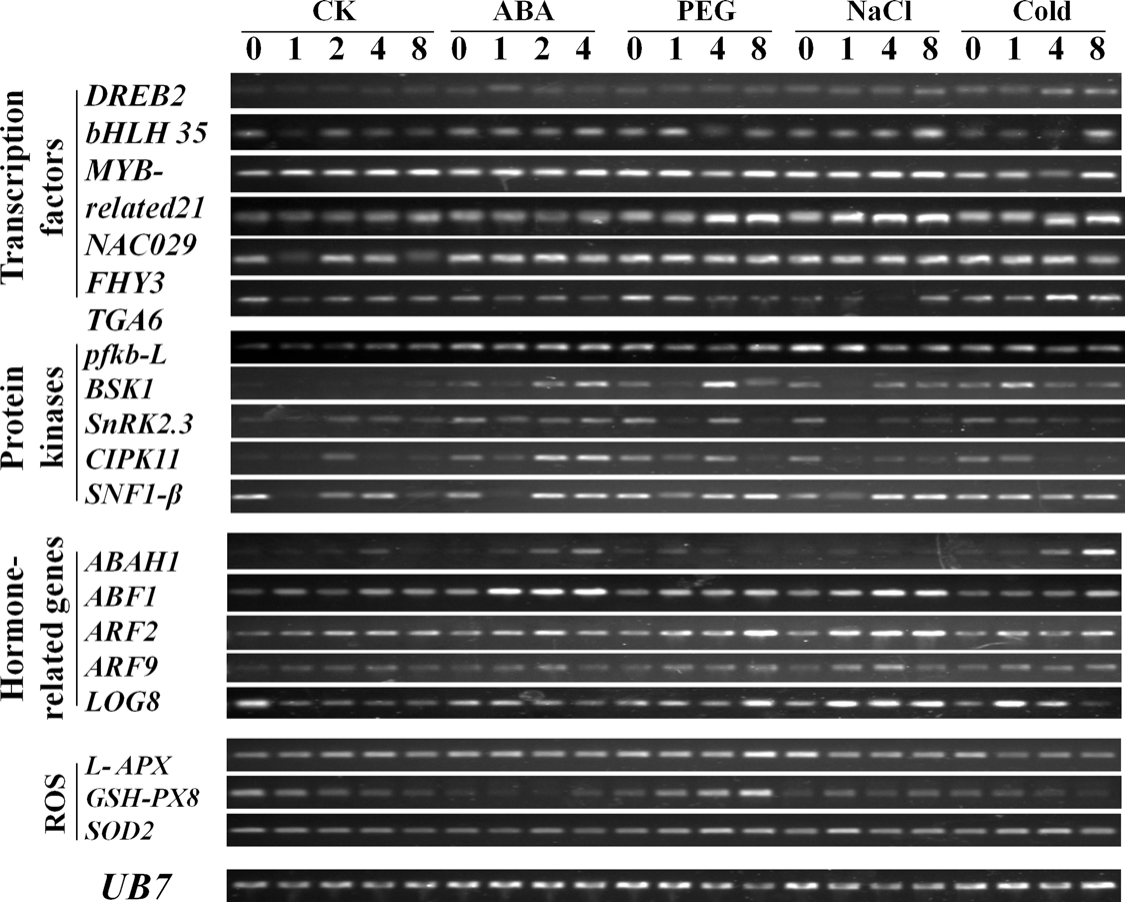
RT-PCR analysis of selected genes under different abiotic stress environments. 0, 1, 2, 4, and 8 refer to samples from different time points (0 h, 1 h, 2 h, 4 h, and 8 h) after treatment by different stress factors (200 mM ABA as ABA, 15% PEG as PEG, 200 mmol L^-1^ NaCl as NaCl and 4°C as cold).

In accordance with previous studies, ABA is a widely studied phytohormone, and its role in ameliorating abiotic stress in plants is well established [39]. There are three major components of ABA signaling: pyrabactin resistance (PYR)/PYR1-like (PYL)/regulatory component of the ABA receptor (RCAR), protein phosphatase 2C (PP2C; a negative regulator) and SNF (sucrose non-fermenting) 1-related protein kinase 2 (SnRK2: a positive regulator). These are gathered as a double negative regulatory system and form a signaling complex known as the ‘ABA signalosome’ [40]. Negative regulation of MAPK (MAPK4 and MAPK6) by *A*. *thaliana* PP2C, AP2C1 also potentially links PP2Cs to cold and drought stress responses [41]. The SnRK2 family members are the key regulators of plant responses to multiple abiotic stresses. SnRK2s (40 kDa) are monomeric serine/threonine protein kinases. Major transcription factor families, which are concerned with the regulation of multiple abiotic stress responses, include *bZIP*, *MYB*, *MYC*, *NAC*, *ERF* and *DREB/CBF* (C repeat binding factor). From all the stress-related genes in the cDNA library, 45 genes, including three genes in the ABA pathway, one *WRKY* gene, one *MYB* and *MYB*-related gene, one *AP2-EREBP* gene, two auxin response factors, one brassinosteroid-regulated family protein, two cytokinin genes, two ethylene-responsive factors, two gibberellin-related genes, one gene responsive to jasmonic acid and salicylic acid, two *JAZ* genes, one *NAC* gene, one *bHLH* gene, one *bZIP* gene, one *FAR1* gene, one LEA family member, 8 redox-related genes, two AMPK family members, two BSK family members, three CDPK family members, one CIPK family member and four SNF1 family members were chosen for validation by RT-PCR. The main results corresponded to the data from the transcription profiles, as depicted in Fig 7. In our results, the expression of *DREB2* is up-regulated in cold treatment after 4 h; *NAC029* is up-regulated in PEG, NaCl and cold treatments; and *TGA6* is down-regulated in PEG and NaCl treatments, but is up-regulated in cold treatment. *CIPK11* is up-regulated in ABA treatment, *GSH-PX8* is up-regulated in PEG treatment, *ABAH1* is up-regulated in ABA and cold treatments, and *ABF1* is especially up-regulated in all the four treatments (ABA, PEG, NaCl and cold).

## Discussion

### Gene enrichment for stress tolerance in cotton

EST sequencing is an effective and comparatively cost-efficient technology for large-scale gene isolation and annotation, genome and physical mapping, discovery of gene expression, molecular marker development, and physical mapping [24,27]. Large-scale sequencing of ESTs has previously been published for cotton, but it was either limited to *G*. *hirsutum* and *G*. *arboreum* [24, 42] or cotton fiber [26, 27,43, 44]. Currently, more than 470,000 ESTs from cotton are deposited in the dbESTs of NCBI Genbank; however, less than 40,000 ESTs are from *G*. *barbadense*, and most of them are not well characterized in response to stress. Due to its low yield, *G*. *barbadense* is less widely cultivated than *G*. *hirsutum* all over the world. Moreover, despite its advanced properties of silkiness, long staples, luster, and marked strength for fiber, *G*. *barbadense* is more sensitive to environmental changes during the growth and development stages. Thus, it is a good material for investigating the stress response in cotton. In this study, we produced large-scale EST sequences from a normalized cDNA library of *G*. *barbadense* Hai-7124 treated with different stress factors (heat, cold, salt, drought, potassium and phosphorus deficit and *Verticillium dahliae* infection). The ESTs were gathered into 3,135 uniESTs and annotated using similarity searches. Similar search was used against the four cotton species EST resources, up to 99.9% (3,132) of the ESTs showed similarity with ESTs from one or more species, and 61.5% (1928) were shared by all four species. However, there were 3 unidentified uniESTs from our library that could be counted as novel genes in *G*. *barbadense*. Using reference genome sequences that were available, uniESTs were mapped to unigenes of different cotton species genomes. A total of 2,845 (90.7%) unigenes showed similarity with one or more species, and 2,718 (86.7%) unigenes were shared by four cotton species.

A large number of cotton uniESTs were related againt responses to biotic and abiotic stresses according to GO analysis for the biological process category (Fig 5C) and Plant Stress Protein Database (PSPDB) annotations (S10 Table). Similarly, many abundant uniESTs encoded stress-responsive proteins, although highly abundant ESTs were normalized in the cDNA library. The annotation results proved that many previously reported stress-related genes were already recorded in these libraries, for example *NHX1*, *MLP*, *DREB*, *ERF*, *HSP*, *POD*, *GST*, *PP2C*, *ERD*, senescence-associated gene, and others. [45–47]. This protocol can be used to isolate other genes involved in cotton response to multiple abiotic stresses (for instance, drought, salt, and cold). The identification of putative stress-related genes would provide a meaningful framework for understanding the sea-land cotton mechanisms. These *G*. *barbadense*-specific sequences could perhaps lead to the differences between *G*. *barbadense* and other cotton species. The openly available ESTs from *G*. *barbadense* will serve as a valuable genomic resource and facilitate further molecular study and breeding of stress-tolerant cotton.

### Transcription regulation of multiple stress responses in cotton

Plants have a molecular mechanisms network that responds to various biotic and abiotic stresses. This network may differ depending on the nature of the biotic and abiotic stress signal. Different response pathways are already explained in plants that regulate responses to miscellaneous environmental signals [48–52]. Combining blast annotations, GO annotations, plant transcription factor database (PlantTFDB) annotations and Plant Stress Protein database (PSPDB) annotations, a huge number of stress-related genes and stress-induced genes are described in the current libraries.

The phytohormones ABA, ET, SA and JA act synergistically or antagonistically to regulate plant responses to pathogens and abiotic stress. ABA plays significant roles in performing as a signal in response to drought, low temperature, osmotic stress and pathogen infection and triggering a quantity of changes in plant physiological and developmental processes, resulting in adaptation to stress conditions [53–55]. In our study, among the 31 hormone-related genes, two auxin response factors (SRS0426, *ARF2* and SRS1821, *ARF9*), one brassinosteroid-regulated family protein (CO000463, *XTH16*), two cytokinin genes (SRS2102, *LOG3* and SRS1232, *LOG8*), two ethylene-responsive factors (SRS2210, *BBM* and SRS1172, *ERF1*), two gibberellin-related genes (CO000098, *GA3* and SRS2432, *GA3OX3*), and one gene responsive to jasmonic acid (SRS1970, *JAZ12*) and salicylic acid (SRS0354) respectively, involved in eight kinds of phytohormones were identified.

The central phenomenon of plants against biotic and abiotic stress is regulatory proteins, including transcription factors and protein kinases [56]. Transcription factors are fundamental to the regulation of cellular pathways in the response to abiotic and biotic stimuli. Under the current study, 464 transcription factor mRNAs were enriched to express over a wide variety of abundances under different stress conditions (Fig 6 and S9 Table). Among these, a subset of transcription factor families were linked to functions in plant response to abiotic stress (*NAC*, *WRKY*, *FHA*, *CBF/DREB*, and etc.), and some of them were present in both biotic and abiotic stresses (*MYB*, *AP2/EREBP*, *bHLH*, and etc.). The enrichment of transcription factors perhaps ameliorated the modulation of cellular redox levels and the evasion or delay of stress-like processes, which would help plants adapt to the stress environment quickly. Cotton contains more than 200 MYB genes, as revealed by the recently released genome sequence of the diploid cotton *Gossypium raimondii* [57]. MYB genes of mostly plants encode proteins of the R2R3-MYB class. An *Arabidopsis* R2R3-type MYB transcription factor, *MYB96*, regulates lateral root meristem activation under drought conditions via ABA-auxin signaling crosstalk [58]. Interactions with bHLH proteins can modulate R2R3-MYB activities. In addition, bHLHs represent the second-largest type of transcription factor in *Arabidopsis* [59]. Currently, there was evidence suggesting that bHLHs can regulate plant responses to multiple abiotic stresses [49]. *GbbHLH35* is homologous to *AtbHLH35*, which responds to cold and jasmonic acid, as found in this study. The bZIP transcription factors were considered to participate in multiple abiotic stress responses and ABA signaling [60].

Protein kinases and phosphatases are indispensable in specialized signaling pathways in which stress signals are identified and transmitted [34]. Among the protein kinases active in stress signal transduction in plants are those shared by all eukaryotic organisms, as in the case of mitogen-activated protein kinases (MAPKs) [61], AMP-activated protein kinases (AMPKs) [62], and plant-specific ones, such as calcium-dependent protein kinases (CDPKs) [63, 64] and the majority of the SNF1-related kinases (SnRKs) [65, 66]. The main protein kinase families identified in the current study are receptor-like protein kinase (RLK), calcineurin B-like calcium sensor interacting protein kinase (CIPK), mitogen-activated protein kinase (MAPK), sucrose-non-fermenting 1(SNF1) kinase, calcium-dependent protein kinase (CDPK), and AMP-activated protein kinase (AMPK), among others (S11 Table). Receptor-like kinase (RLK) proteins play an essential part in signal transduction and contribute in a wide extent of processes in response to plant hormones and environmental aspects [67]. In *Arabidopsis*, a receptor-like kinase gene (*GbRLK*) from *Gossypium barbadense* boosts salinity and drought-stress tolerance [68]. Overexpression of a CIPK gene from cotton, *GhCIPK6*, significantly enhances tolerance to drought, salt and ABA stresses in transgenic *Arabidopsis* [69]. Regulations are made to the guard cell anion channel *SLAC1* by CDPK protein kinases assisted by distinct Ca2^+^ affinities. The calcium-dependent (*CPK21*) and -independent branch (*OST1/CPK23*) of ABA signal transduction in guard cells seem to converge at the level of SLAC1-ABI1 regulation [70]. SnRKs, including SNF1 kinases from yeast and AMPKs from mammals, are positive regulators of ABA-mediated signaling, thus shouldering the responsibility of the phosphorylation of bZIP transcription factors known to bind cis-regulatory motifs with ACGT as a core sequence [71].

In addition, cellular oxidation is of importance in all abiotic and biotic stress responses, and plant cells generally stand up to the chanllenges of high rates of generation of superoxide, H_2_O_2_, and even singlet oxygen [72]. ROS modulate plant responses to diverse environmental signals [38, 73, 74]. The classification of assigned protein motif/domain for ESTs and unigenes indicated that ESTs encoding redoxin-like thioredoxin, ferredoxin, POD, glutathione S transferase (GST), SOD and catalase (CAT) were enriched in the present library (S13 Table). There are approximately 321 ESTs with the GO annotation of ‘oxidation reduction’ at level 4 of the category Biological Process. APX1 plays a major role in regulating immune responses, and S-nitrosylation at Cys-32 strengthens its enzymatic activity of scavenging hydrogen peroxide, ending up with increased resistance to oxidative stress [75]. Mutation of *AtGSTU17* showed more tolerance to drought and salt stresses in contrast to that in wild-type plants [76].

Studies also point out a better coordination of plant responses to biotic and abiotic stresses, embodying the activation/ suppression of overlapping sets of genes in response to biotic and abiotic stresses [77–79]. Negative regulation of MAPK (MAPK4 and MAPK6) by *PP2C*, *AP2C1* also potentially links *PP2C*s with cold and drought stress responses [41]. ROS-induced activation of MAPKs stands at the central stage for intermediating cellular responses to multiple stresses [37]. *Arabidopsis OXIDATIVE SIGNAL-INDUCIBLE1* (*OXI1*), which encodes a putative serine/threonine kinase, regulates the activation of MPK3 and MPK6 by ROS and is also entailed for pathogen resistance [80]. As a result, plant responses to environmental signals share principal regulatory mechanisms with complex interactions between responses to plant hormones, pathogens, abiotic stresses and ROS.

## Materials and Methods

### Plant materials, growth conditions and stress treatments

The cotton cultivar *G*. *barbadense* Hai-7124 was used for all the experimental work. After pregermination, the seeds were grown in sand containing vermiculite under green house environment with a 16 h light/8 h dark cycle at 28˚C and 50–60% humidity. Different abiotic stresses, heat (40°C), cold (4°C), 200 µM NaCl, and drought as examples, were imposed on three-week-old seedlings at approximately the 3-leaves stage. Heat and cold treatments were conducted in the plant growth room at 40°C or 4°C for one or two days. Salt treatment was conducted by watering three-week-old seedlings with 200 µM NaCl. Drought treatment was imposed on three-week-old seedling grown under normal conditions by withholding water for approximately 7 days until the soil moisture was below 50%. A hydroponics experiment was also conducted for the nutrient deficiency treatment with complete Hoagland solution culture [81]. The potassium and phosphorus deficiency treatment was conducted using incomplete Hoagland solution culture with three-week-old seedlings at approximately the 3-leaves stage. *Verticillium dahliae* treatment was conducted by inoculating the damaged cotton root with the strain *V991* (Cotton *verticillium dahliae* wilt fungus) in three-week-old seedlings [82]. Meanwhile, three-week-old seedlings grown under normal conditions were the controls. The experiments were conducted in three biological replicates, and each replicate represented 20 seedlings. The leaves and roots of treated and control plants were harvested at different time intervals after treatments (time point of sampling: heat at 1, 2, and 4 h; cold, 1, 2, 4, and 8 h; NaCl, 1, 2, 4, 8, and 12 h; drought, 1, 6, 12, 24, and 48 h; potassium and phosphorus deficit at 1, 3, 6, 12, and 24 h; *Verticillium dahliae* infection, 0, 1, 2, 4, 8, 12, 24, 48, 72, and 96 h), crushed in liquid nitrogen and stored at -70˚C for later use.

### Individual and normalized cDNA library construction

A modified guanidine thiocyanate method was utilized to extract total RNA from the collected samples [83]. The Oligotex mRNA kit (cat. no. 70042, Qiagen, Germany) was used to isolate and purify polyA mRNA, following the manufacturer’s protocol using a mixture containing an equal amount of total RNA from different time points. Individual cDNA libraries were constructed using Invitrogen CloneMiner^TM^ cDNA library construction kits (cat. no. A11180, Invitrogene, USA) according to Tu et al. [40]. Biotin-attB2-oligo (dT) primer was used to synthesize the first strand cDNA. Double-strand cDNA was bound to attB1 adapter and then cDNA-size fractionation columns were used to purify it. The BP recombination reaction between the attB-flanked cDNAs (>500 bp) and attP-containing donor vector pDONRTM222 was performed, and the products were transformed into ElectroMAXTM DH10BTMT1 Phage Resistant Cells to generate Gateway cDNA libraries. Colonies were randomly selected from the plating assay plates. Plasmid DNA was extracted, and the cDNA library was confirmed by plasmid digestion. The normalized library was developed using saturation hybridization between genomic DNA and mixed plasmid DNA from individual cDNA libraries [43].

### EST sequencing and processing

Randomly selected clones were shifted into 384-well plates. Plasmids were isolated from randomly selected clones of the normalized cDNA library. Single-pass sequencing from the 5’ end was performed with T7 promoter primers with the Sanger method using an ABI 3730 xl automatic DNA sequencer (Auke Biotech Co., Ltd., Beijing, China). High-quality nucleotide bases were called using the Phred program [84, 85], while low-quality bases (Q20, 99% accuracy) were removed from the sequence ends. These sequences were then filtered to remove vectors, repeats, and contaminating sequences using the PESTAS web server for EST analysis and sequence mining [86]. Finally, sequences shorter than 100 bp and those including only homo-polymer tracks of more than 20 nucleotides such as polyA20 and polyT20 were removed. To generate the uniEST set, the filtered ESTs were assembled into contigs and singletons (uniESTs) using the CAP3 program (http://seq.cs.iastate.edu/) with 98% sequence identity and a 40-bp minimum match length.

### Annotation and functional classification

To annotate uniESTs, a BLAST search was carried out to find the resemblance between the ESTs and sequences deposited in public databases. Primarily the uniESTs were mapped to several reference sequences, i.e., ESTs of different cotton species downloaded from GenBank (*G*. *hirsutum*, 337,811; *G*. *arboreum*, 64,798; *G*. *raimondii*, 63,577; and *G*. *barbadense*, 39,115; (http://www.ncbi.nlm.nih.gov/dbEST/dbEST_summary.html)) and several reference genome sequences, i.e., unigenes from the cotton genome of different cotton species (*G*. *arboreum*, 75,594 (https://www.cottongen.org/); *G*. *raimondii*, 77, 267 (https://www.cottongen.org/); *G*. *hirsutum*, 288,299 (https://www.cottongen.org/); *G*. *barbadense*, 80,876 (http://cotton.cropdb.org/cotton/index.php)).

In addition, the uniESTs were further searched in the NCBI non-redundant (nr) protein database (http://blast.ncbi.nlm.nih.gov/ Blast.cgi) using the blastx program and the protein sequences of *Arabidopsis* (http://www.arabidopsis.org/Blast/index.jsp), *Oryza sativa* (http://rice.plantbiology.msu.edu/analyses_search_blast.shtml), *Populus trichocarpa* (http://genome.jgi-psf.org/poplar/), and *Vitis vinifera* (http://www.genoscope.cns.fr/externe/) using blastx (E-value ≤ 10^−5^). SimiTri program was used to display the relative similarity relationships between *G*. *barbadense* and other species [29].

In addition, Blast2GO program was used to perform the functional annotation of the uniEST set using GO terms (www.geneontology.org) [87]. This programe was used with default parameters, based on blastx against the NCBI non-redundant (nr) protein database. Finally, analysis of the biological processes/pathways was carried out using KOBAS2.0 (KEGG Orthology Based Annotation System) [31].

### Identification of putative transcription factors and stress-related genes

Blastx was used against a comprehensive plant transcription factor database (PlantTFDB, http://plntfdb.bio.uni-potsdam.de/v3.0/) to identify the putative transcription factors [33]. UniESTs with homology to genes involved in the plant stress response were identified by blastx searches against the Plant Stress Protein database (PSPDB, http://www.bioclues.org/pspdb/) [88].

### RT-PCR analysis

The uniESTs related to stress response genes identified from the cDNA library were selected for validation by reverse transcriptase-polymerase chain reaction (RT-PCR). According to the cDNA sequences, gene specific primers (as shown in the S14 Table) were designed by using Primer Premier 5.0 (http://www.premierbiosoft.com/crm/jsp/com/pbi/crm/clientside/ProductList.jsp) and synthesized commercially (Genscript Bioscience, Nanjing, China). Superscript III Reverse Transcriptase (Invitrogen, Carlsbad, CA) was used to generate first-strand cDNA from 5 μg of RNA samples in accordance with the manufacturer’s instructions. Standard PCR system was as follows: denaturation at 94°C for 5 min, followed by 28–35 cycles at 94°C for 30 s, 58–60°C for 30 s, and 72°C for 30 s and a final step at 72°C for 7 min. The cotton UBQ7 gene with GenBank accession number: DQ116441was used as an internal control. Agrose gel (0.8%) having ethidium bromide, were used to separate 10 µl of PCR products. Each RT-PCR analysis was repeated three times.

## Supporting Information

S1 Fig The length distribution of ESTs before (1A) and after (1B) trimming vectors. (TIF)

S2 Fig The third-level GO terms for cellular component (2A), molecular function (2B), and biological process (2B). (TIF)

S1 Table Individual and normalized cDNA libraries for *G*. *barbadense* cv. Hai-7124 treated with different stress factors.
(XLSX)

S2 Table Distribution of insert size in the normalized cDNA library. (XLSX)

S3 Table Thirty highly abundant genes among the 6,047 ESTs. (XLSX)

S4 Table Details of the 3,135 uniEST blastx search against ESTs of four cotton species from GenBank (E-value ≤ 10^−5^). S4-1: *G*. *hirsutum*; S4-2: *G*. *arboreum*; S4-3: *G*. *raimondii*; and S4-4: *G*. *barbadense*.
(XLSX)

S5 Table Details of the 3,135 uniEST blastx search against unigenes from the genome of different cotton species (E-value ≤ 10^−5^). **S5-1:** *G*. *arboreum* (https://www.cottongen.org/); **S5-2:** *G*. *raimondii* (https://www.cottongen.org/); **S5-3:** *G*. *hirsutum* (https://www.cottongen.org/); and **S5-4:** *G*. *barbadense* (http://cotton.cropdb.org/cotton/index.php).
(XLSX)

S6 Table Details of the 3,135 uniEST blastx search against the non-redundant (nr) protein sequence database in GenBank. A total of 2,820 (90.0%) unigenes showed significant hits (E-value ≤ 10^−5^).
(XLSX)

S7 Table Details of the 3,135 uniEST blastx search against the protein sequences of *Arabidopsis* (S7-1), *Oryza sativa* (S7-2), *Populus trichocarpa* (S7-3), and *Vitis vinifera* (S7-4) (E-value ≤ 10^−5^). (XLSX)

S8 Table The summary of GO function categories of all uniESTs. S8-1: Summary of GO function categories of all uniESTs; the three GO categories biological process (level 4) (S8-2), molecular function (level 4) (S8-3), and cellular component (level 4) (S8-4) are presented.
(XLSX)

S9 Table Categories of putative transcription factors. The 3,135 uniESTs were identified by a blastx search against PlantTFDB (http://plntfdb.bio.uni-potsdam.de/v3.0/) to identify putative TFs. S9-1: 464 sequences (14.8% of uniESTs) had homologs in PlantTFDB (E-value ≤ 10–5); S9-2: the most abundant putative transcriptional factors. (XLS); S9-3: the distribution of TF families in five related species.
(XLSX)

S10 Table Classification of stress-related genes. **The 3135 uniESTs were identified by a blastx search against the Plant Stress Protein database (PSPDB, http://www.bioclues.org/pspdb/) to identify stress-related genes. A total of 919 sequences had homologs (blastx, E-value ≤ 10^−5^) in the PSPDB**.
(XLSX)

S11 Table The main protein kinase families in stress-related genes. (XLSX)

S12 Table The distribution of hormone responsive genes. (XLSX)

S13 Table The distribution of active oxygen scavenging enzymes. (XLSX)

S14 Table Gene-specific primers used for RT-PCR. (XLSX)
