# Supplementary figures and images for: Identification of Multiple Stress Responsive Genes by Sequencing a Normalized cDNA Library from Sea-Land Cotton (Gossypium barbadense L.)
Source: PLoS One. 2016 Mar 31;11(3):e0152927. doi: 10.1371/journal.pone.0152927 (PMC4816313; doi:10.1371/journal.pone.0152927)

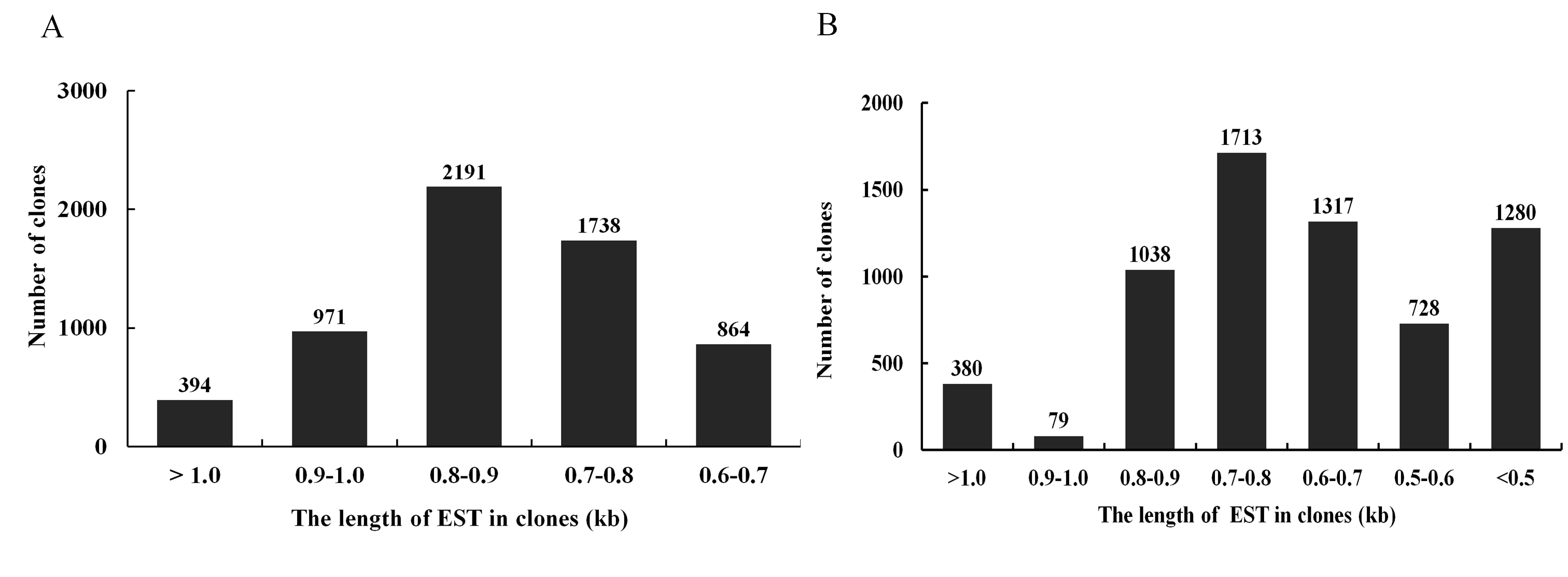

Supplement: S1 Fig — (TIF) [file pone.0152927.s001.tif]

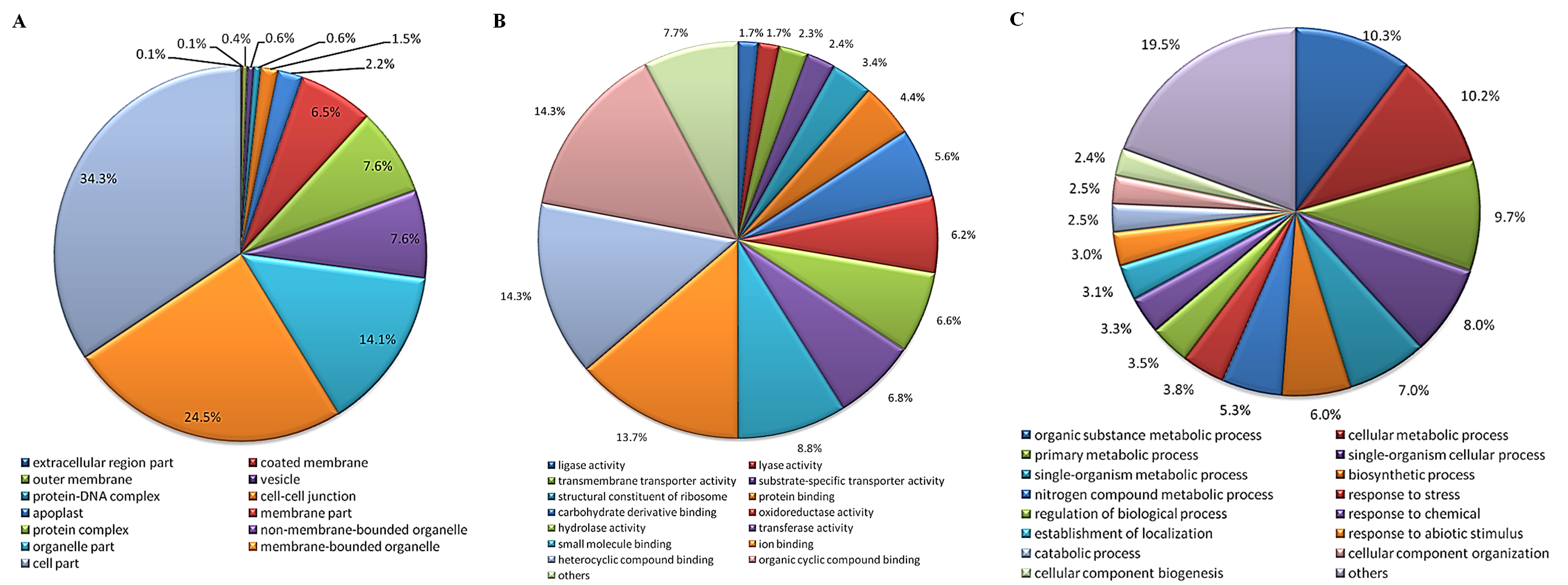

Supplement: S2 Fig — (TIF) [file pone.0152927.s002.tif]
